# Retrospective evaluation of percutaneous 3D-navigated screw fixation for fragility fractures of the sacrum: technical notes and four-year experience

**DOI:** 10.1038/s41598-023-39165-8

**Published:** 2023-07-28

**Authors:** Andreas Kramer, Martin Naisan, Stefan Kindel, Marcus Richter, Florian Ringel, Philipp Hartung

**Affiliations:** 1 Department of Neurosurgery, University Medical Center Mainz, Langenbeckstraße. 1, 55131 Mainz, Germany; 2 Spine Center, St.-Josefs Hospital, Wiesbaden, Germany

**Keywords:** Medical research, Risk factors, Trauma

## Abstract

The incidence of fragility fractures of the sacrum is increasing due to demographic changes. In this study, we introduce the 3D-navigated monoportal percutaneous sacroiliac screw fixation (PSS) as a technical advancement for treating fragility fractures of the sacrum. We included all patients who underwent the 3D-navigated monoportal PSS for fragility fractures of the sacrum. The fractures were classified using the Fragility Fractures of the Pelvis score (FFP). We provide a step-by-step illustration of the surgical technique. The objective of this study was to assess the feasibility and safety of the investigated technique. Forty-six patients (36 female, 10 male) with a median age of 81.5 years were included in the study. The fracture classification revealed 23 FFP2 (50%), 5 FFP3 (11%), and 18 FFP4 (39%) fractures. In 35 cases (76%), only transsacral screws were implanted in S1 and S2, with an average incision-to-suture time of 52.6 min. The remaining eleven patients underwent additional anterior pelvic ring fixation, lumbar instrumentation, or kyphoplasty. There were no instances of nerve root, vascular, or pelvic organ injuries. The median postoperative in-hospital stay was six days. Out of the 36 patients who were followed up, four patients required revision surgery due to screw loosening. No significant risk factor for screw loosening was identified in the multiple regression analysis. The presented monoportal PSS technique for fragility fractures of the sacrum is a promising minimally invasive approach with a low complication rate and excellent short-term outcomes.

## Introduction

The incidence of age-related diseases, including fragility fractures of the pelvis, is on the rise due to demographic changes and an aging population^1,2^. Unlike high-energy pelvic fractures in younger individuals that can lead to severe blood loss and pelvic organ injuries, geriatric population may sustain pelvic ring fractures with minor trauma^3–6^. Concomitant hemorrhage or pelvic organ injuries are rare; however, these injuries often result in debilitating back pain. Therefore, the primary treatment goal for this patient group is to restore functionality and prevent immobility, which can lead to complications such as ulcers, skeletal demineralization, and muscle atrophy. Various osteosynthesis procedures are available depending on fracture morphology, instability degree, and pelvic anatomy. Given the age and comorbidities commonly found in the geriatric population, minimally invasive techniques with shorter incision-to-suture time and reduced soft tissue trauma are preferred over conventional techniques^7^. Additionally, the choice of osteosynthesis technique and materials is crucial due to the frequently encountered osteoporotic and poor-quality bone in this population^8,9^. The use of 3D navigation has shown improved accuracy in screw placement compared to 2D navigation or fluoroscopic control^10^. Any potential drawbacks, such as increased surgical time due to intraoperative imaging and computer navigation, can be mitigated by routine utilization of these technical devices with an optimized workflow.

Fragility fractures of the sacrum commonly involve both lateral masses of the sacrum and often coincide with fractures of the anterior pelvic ring. Therefore, a bilateral osteosynthesis is necessary for operative treatment of these fractures. Traditionally, a biportal percutaneous sacroiliac screw (PSS) fixation technique at the S1 and/or S2 level is chosen as minimal invasive approach. However, biomechanical finite element model studies have demonstrated that transsacral screws provide superior stability compared to bidirectional sacroiliac screws for both unilateral and bilateral sacral fractures^11,12^. Furthermore, 3D statistical models have identified low bone mass in the sacral vertebral bodies of patients with fragility fractures of the sacrum, suggesting that transsacral osteosynthesis procedures with improved screw-bone contact may be advantageous^8,13^. Clinical data evaluating the feasibility and perioperative complication rate of 3D-navigated monoportally inserted transsacral screws is limited, although several single-center studies have reported promising results^14–16^.

The preliminary hypothesis of this study was that the 3D-navigated monoportal percutaneous sacroiliac screw fixation (PSS) technique is a safe and effective minimally invasive approach for treating fragility fractures of the sacrum, with low complication rates and favorable short-term outcomes. To investigate this, we conducted a retrospective study of our four-year experience with this technique and provided a step-by-step description of the procedure. Relevant surgical parameters, including screw positioning accuracy and perioperative complication rates, are evaluated.

## Material and methods

### Ethical approval

The ethical committee of the Medical Association of Hessen, under research no. 2021- 2558-evBO, approved this study. All surgical procedures were conducted in accordance with Good Clinical Practice Guidelines. Informed consent was obtained from all subjects and/or their legal guardian(s).

### Patient selection and data collection

**Figure 1:**
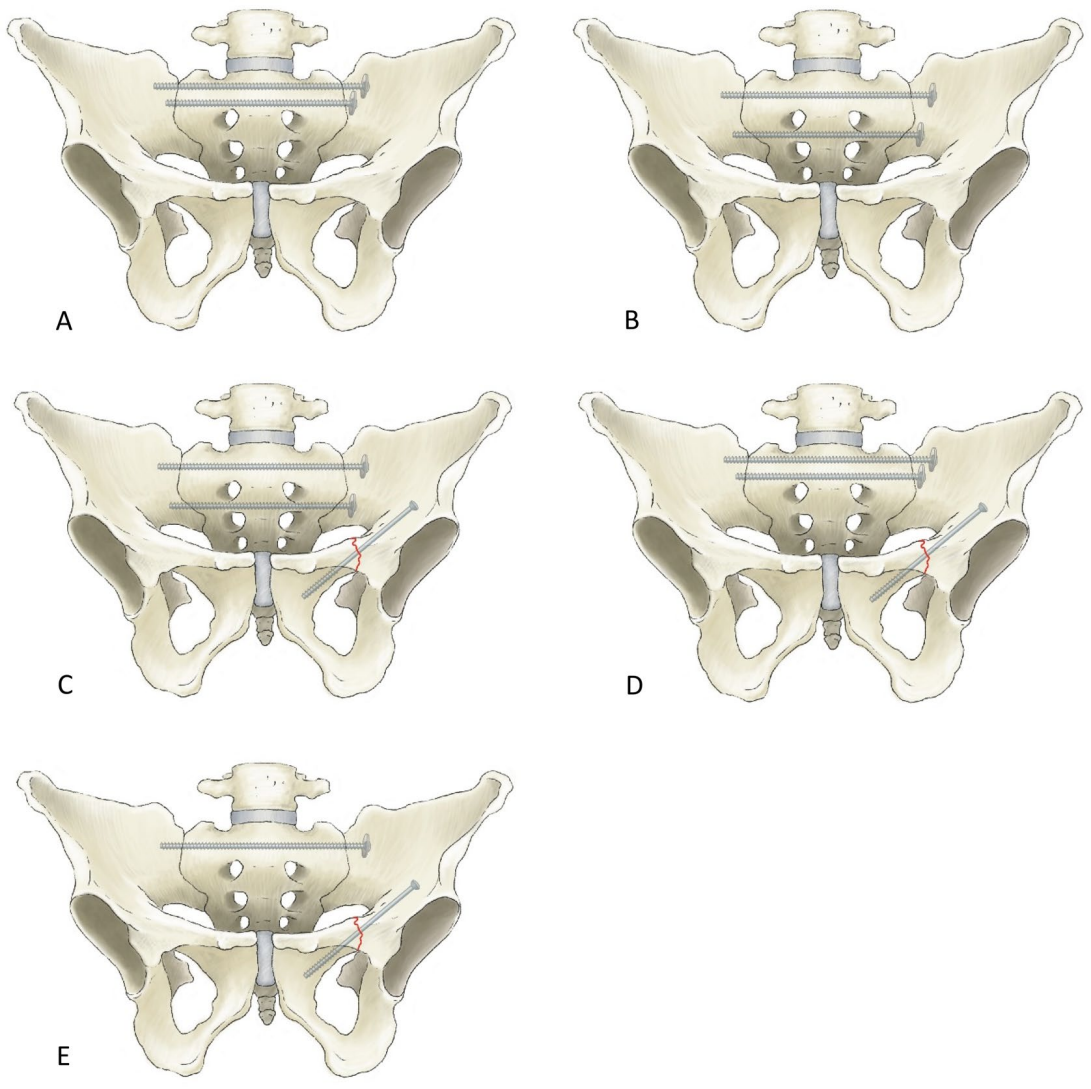
Illustration of different options for monoportal transsacral screw placement. (**A**) Two transsacral fully threaded screws at S1 level; (**B**) Two transsacral fully threaded screws at S1 and S2 level; (**C**) Two transsacral fully threaded screws at S1 and S2 level plus one partially threaded screw through the superior pubic ramus; (**D**) Two transsacral fully threaded screws at S1 level plus one partially threaded screw through the superior pubic ramus; (**E**) One transsacral fully threaded screw at S1 level plus one partially threaded screw through the superior pubic ramus.

In this retrospective cohort study, we examined the data of all patients who underwent 3D-navigated monoportal osteosynthesis as a treatment for low- energy fractures of the sacrum between 2018 and 2021. Overall, 124 patients underwent operative treatment for fractures of the posterior and/or anterior pelvic ring. Among them, 46 patients received treatment with extended sacro-iliac screws and were therefore included in this study. Patients who underwent additional screw fixation for a fracture in the anterior pelvic ring were also considered. Table 1 provides an overview of the patients' demographics and fracture configuration. Figure 1 illustrates the possible screw positions based on fracture configuration and individual sacral anatomy. The indications for surgical treatment were immobility and a pain level ≥ 5 despite adequate analgesics, as measured on the visual analogue scale. Exclusion criteria for 77 patients included the use of other osteosynthesis techniques for low-energy fractures, a history of a high-energy trauma, tumorous pelvic lesions, and combination with complex surgical stabilization procedures. Fractures were graded according the Fragility Fractures of the Pelvic Ring (FFP) classification, based on preoperative routine MRI and CT imaging^17^.

**Table 1:** Demographics and fracture characteristics of the patients, presented by categories, with absolute numbers and percentages.

Variable	Category	Number	Percentage
Age	60–69	5	11
	70–79	13	28
	80–89	26	57
	90–99	2	4
Sex	Male	10	22
	Female	36	78
Trauma	Low energy	32	68
	No trauma	14	32
FFP Classification	FFP1	0	0
	FFP2	23	50
	FFP3	5	11
	FFP4	18	39
Anterior pelvic ring fracture	None	13	28
	Ipsilateral	31	67.5
	Contralateral	0	0
	Bilateral	2	4.5
Posterior pelvic ring fracture	Unilateral	13	28
	Bilateral	33	72
Comminuted fracture	Yes	13	28
	No	33	72
H-fracture	Yes	14	30
	No	32	70

Analyzing the records of all patients, the pre- and postoperative clinical status was evaluated based on mobility (immobile, mobile to sitting, mobile with crutches, mobile with walker). The incision-to-suture time was measured, although due to the minimally invasive nature of the technique and negligible blood loss, blood loss could not be quantified. Surgical reports, documentation of the postoperative course, discharge letters, and postoperative radiographic data were examined to identify any surgery-related complications, including nerve root injury, vessel injury, pelvic organ injury, postoperative hematoma, wound healing disorders, surgical site infection, and screw malplacement.

### Surgical procedure

**Figure 2:**
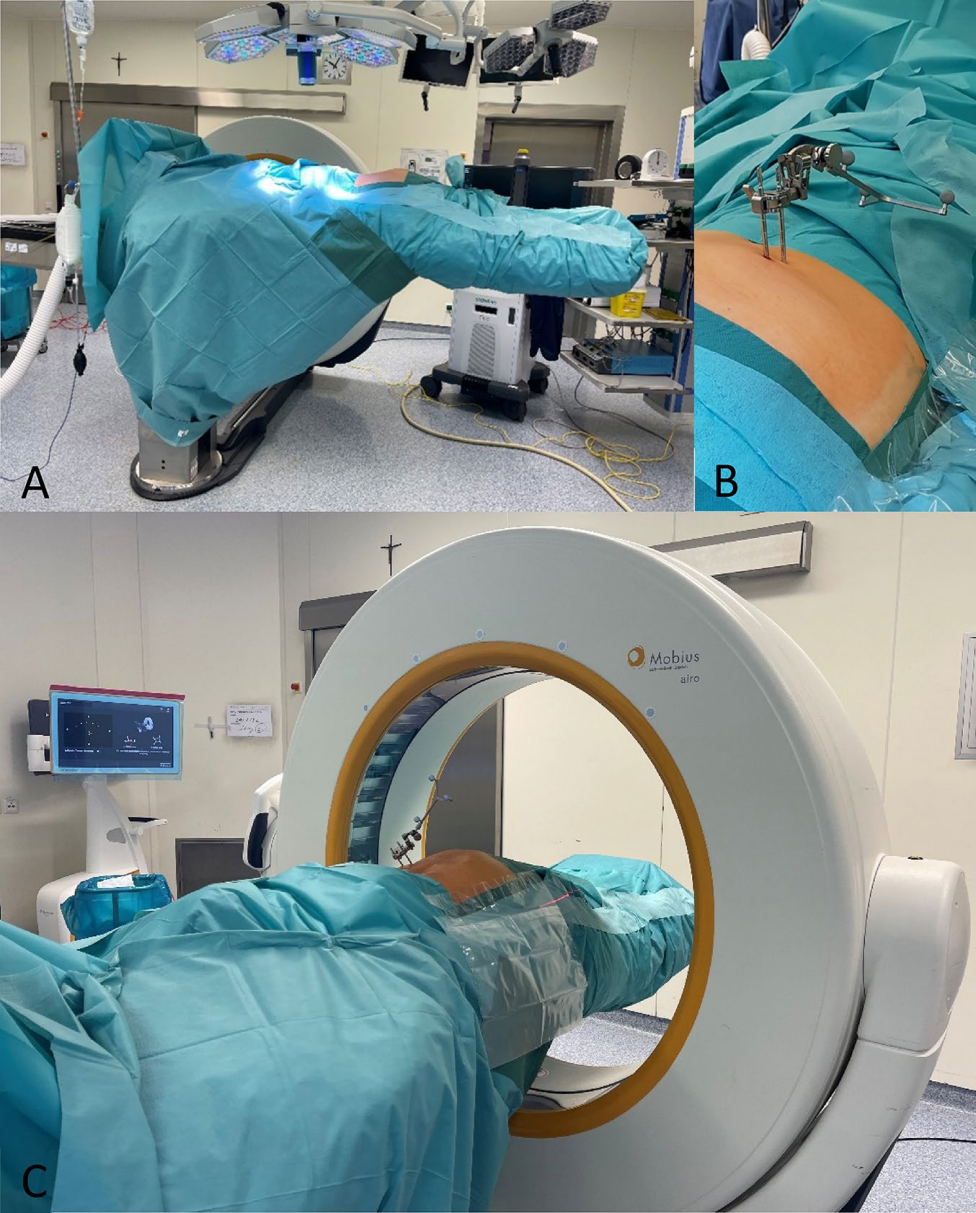
(**A**) 360° sterile draping. (**B**) Navigation tracking device fixed via two pins inserted in the iliac crest. (**C**) After counter-clockwise rotation of the operating table the 3D dataset is acquired using the iCT.

The patient is placed in the prone position on the positioning block under general anesthesia. The arms are positioned with 90° abduction of the shoulder joints and 90° flexion of the elbows cranially. Disinfection is carried out over the entire dorsal and lateral pelvis. Circumferential sterile draping ensures 360° sterility up to the operating table column (Fig. 2).

After a team time-out, the posterior superior iliac spine and the iliac crest are palpated unilaterally. Two fixation pins are then drilled into the iliac crest via stab incisions, to which the navigation tracking device (Brainlab AG, Munich, Germany) is attached.

To generate the 3D data set, the AIRO^®^ iCT (Stryker, Kalamazoo, Michigan, USA) is used. CT parameters and patient data are set in preparation. Laser guidance is employed to determine the extent of the surgical field. After the staff leaves the operating room, the in-house radiological-technical assistant obtains a CT AP scout of the pelvis, covering both ossa ilia and the sacrum. The iCT is performed under temporary apnea after preoxygenation of the patient, to avoid motion artefacts. The acquired data is verified and transferred to the institutional DICOM server and the navigation system (Brainlab Spinal Navigation Software Version 3.0, Brainlab Curve™, Brainlab AG, Munich, Germany). The OR table is then rotated 90° clockwise back to the working position. Following a fresh sterile covering of the floor-facing operating table and a glove change, the accuracy of the virtual reality is checked by aligning the navigation pointer and the fixation pins.

**Figure 3:**
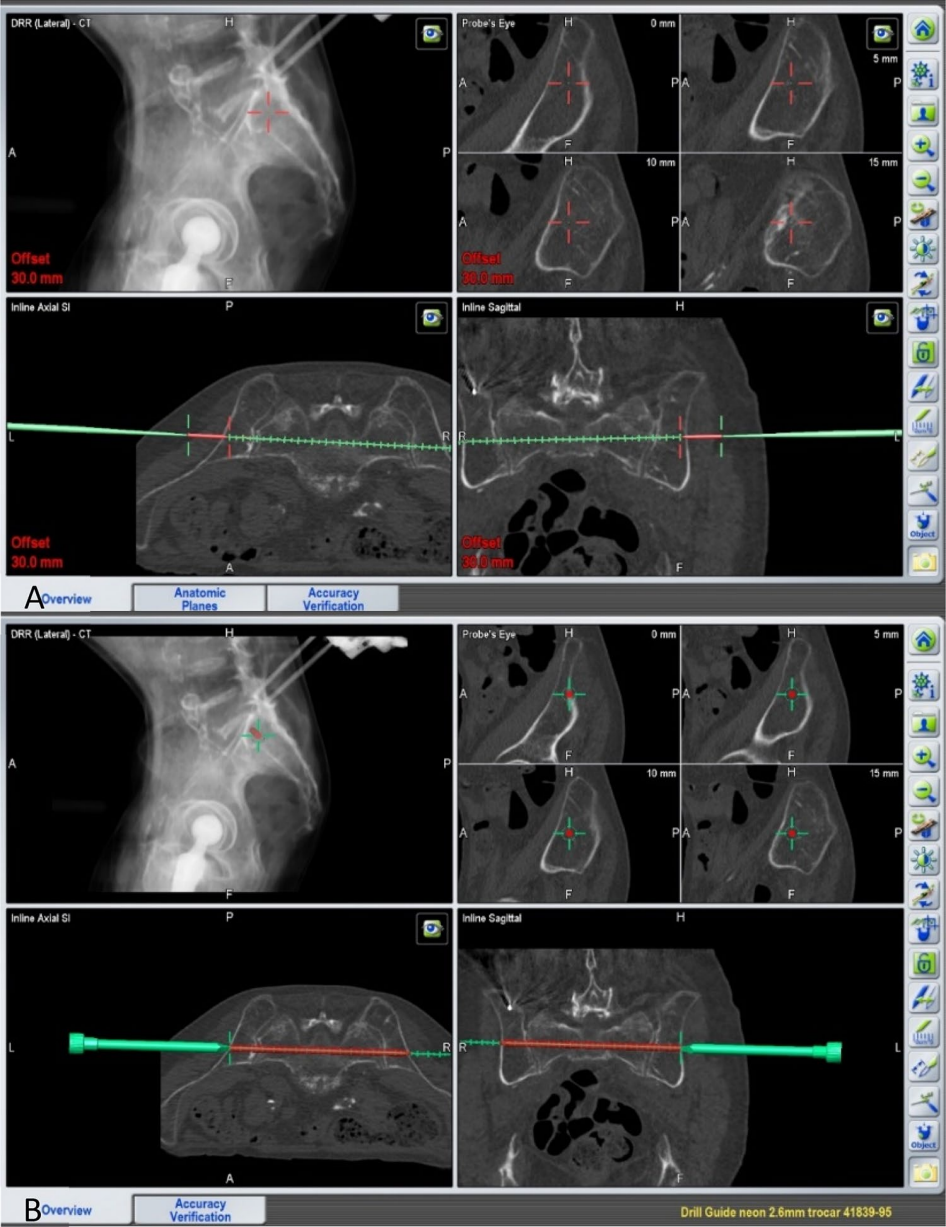
(**A**) Planning the trajectory and site of skin incision using the navigation pointer on skin surface. (**B**) Planning the screw to be implanted using the navigated drill guide.

To plan the incisions, the trajectories of the screws to be implanted are determined at the skin level using the offset setting (Fig. 3A).

**Figure 4:**
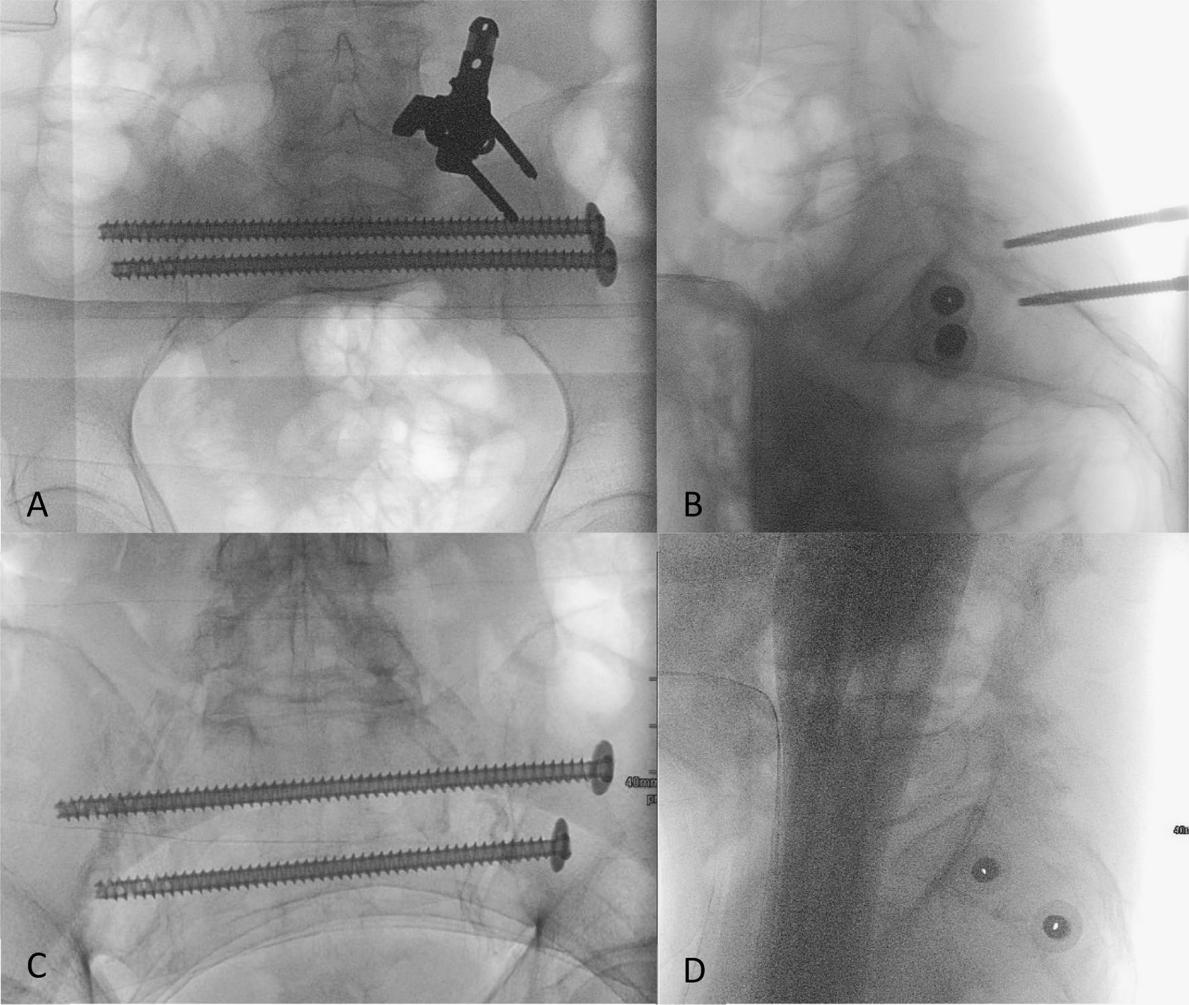
(**A,B**) AP and lateral view of two screws implanted in S1. (**C,D**) AP and lateral view of two screws implanted in S1 and S2.

After a skin incision of approximately 3 cm, blunt dissection is performed onto the cortex of the os ilium. The navigation instruments are registered, and a drill sleeve is inserted at the screw entry point on the ilium. Figure 3B illustrates the planning of a screw starting within the ilium and running through the sacrum and the corridor above the S1 foramina to the opposite side, bridging the contralateral sacroiliac joint. A navigated 2.6 mm drill wire is then inserted through the navigated sleeve and drilled according to the plan. A large-fragment fully threaded screw (TIS™ Königsee, Weye, Germany) of appropriate length, 7.5 mm in diameter and with a washer, is implanted over this wire. Depending on the fracture configuration and the width of the S1 corridor, a second screw is implanted in a similar manner, either caudal to the first screw at the level of S1 or through the corridor between S1 and S2 foramina in S2. The correct positioning of the screws is then documented fluoroscopy in the AP and lateral as well as in the inlet and outlet projections (Fig. 4).

In case of an additional fracture of the anterior pelvic ring requiring treatment, another navigated 2.6 mm drill wire can be placed through the superior pubic ramus into the pubic bone, using the same skin incision. To verify the positioning of the navigated drill wire, the iCT is repeated and, once the correct wire position is documented, a partially threaded screw (TIS™ Königsee, Weye, Germany) is implanted according to the planned length. Following the flushing of the surgical field with sterile saline solution, the wound is closed using subcutaneous and skin sutures.

### Feasibility

Primary objective of this study was to assess the feasibility of the presented technique. Therefore, the aforementioned parameters were collected to analyze the peri- and postoperative complication profile and the clinical status of the patients.

Statistical analysis was conducted using IBM SPSS (IBM, USA, Version 27). Statistical significance was set at an error probability of p ≤ 0.05. Multiple regression analysis was performed using ANOVA to examine risk factors related to screw loosening.

## Results

Of the 46 patients included in this study, ten were male (22%) and 36 (78%) were female. Median age was 81.5 years, ranging from 60 to 91 years.

**Figure 5:**
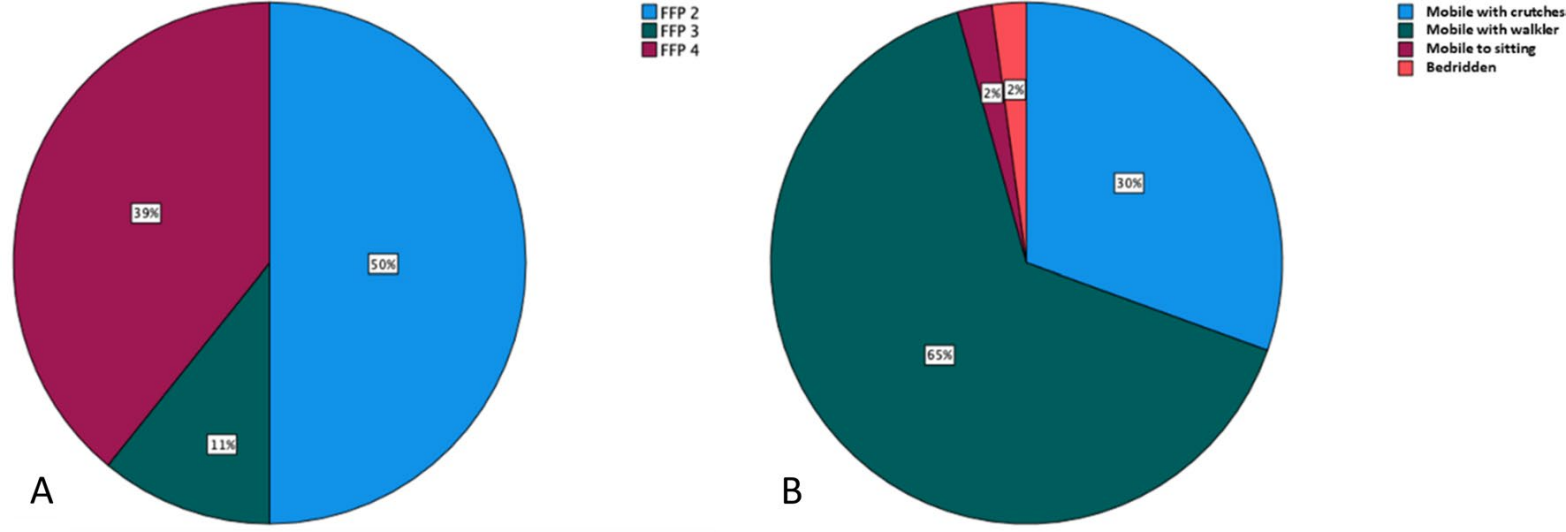
(**A**) Grading of fractures according to FFP classification; (**B**) Mobility at discharge.

In 32 cases (68%), a low-energy trauma caused the fracture, while in 14 cases (32%), no trauma was reported in the medical history. All patients experienced immobilizing pain (visual analogue scale ≥ 5) despite receiving adequate analgesics. No case presented with newly diagnosed neurological deficits. The preoperative grading of fractures using the FFP classification revealed a heterogeneous distribution of fracture types in our collective, as shown in Fig. 5. Table 1 provides an overview of the patients' demographics and fracture characteristics.

The technique of monoportal osteosynthesis was successfully applied in all cases as planned preoperatively. Regardless of the patients’ body size or BMI, a single iCT scan was sufficient to visualize the entire pelvis and enable the planning of screw trajectories required for osteosynthesis of the posterior and/or anterior pelvic ring in every case.

In 35 cases (77%), two transsacral screws were planned and implanted. In six cases (13%), an additional partially threaded screw was used to treat a coexisting fracture of the anterior pelvic ring, and in five other cases, additional surgical procedures (kyphoplasty, lumbar instrumentation) were necessary.

As mentioned earlier, the quantification of intraoperative blood loss was not possible due to the minimal amount of bleeding, which did not require the use of a suction device.

The relevant surgical and anesthesiologic reports, as well as the documentation of the postoperative course and outpatient follow-up examinations, were reviewed for intraoperative and perioperative complications. No perioperative nerve root injury or postoperative neurological deficit were observed. Additionally, there were no incidents of vasculature or pelvic organ injury in the investigated cohort. Postoperative hematoma or wound healing disorders requiring revision surgery were also not encountered. In summary, no complications were attributed to the surgical technique used.

The mean incision-to-suture time required for implanting two screws in S1 or two screws in S1 and S2, respectively, was 52.6 min (SD 13 min). In cases where an additional screw was planned and implanted in the pubic bone, the mean incision-to-suture time was 71 min (SD 13.4 min).

Whether both screws were implanted in S1 or if a second screw at the level of S2 was necessary depended on individual anatomical factors and the intraoperative situation after the first screw was implanted.

Postoperative control of screw placement through ap, inlet, outlet, and lateral X-rays of the pelvis revealed correct positioning of all implanted screws, eliminating the need for any corrective measures.

On the first postoperative day, all patients, except one who remained bedridden, were mobilized under physiotherapeutic supervision. To prevent the risk of stumbling and sudden weight-bearing, initial mobilization was performed using a walker or crutches, with full body weight being gradually induced. At discharge, 14 patients (30%) were mobile with crutches, 30 patients (65%) used a walker, one patient (2%) was able to sit, and one patient (2%) remained bedridden. This resulted in a 98% improvement in mobility compared to the preoperative status.

The median length of postoperative hospital stay was 6 days, ranging of 3–30 days (excluding one outlier who required inpatient treatment for Covid-19 infection under isolation conditions). Patients were routinely discharged to inpatient geriatric follow-up treatment for further clinical and functional consolidation.

Out of the initial 46 patients, 36 attended the scheduled follow-up examinations three and twelve months postoperatively, resulting in a follow up rate of 78%. X-ray examinations of these patients revealed radiological signs of screw loosening, which was confirmed in subsequent CT scans of the pelvis in seven cases (19%).Among these cases, four patients required reoperation, resulting in a reoperation rate of 11% in relation to the followed cohort. In two cases, the removal of the loosened screw was sufficient as fracture healing was complete. In one case, the loosened screw was replaced with a thicker hydroxyapatite coated screw (SI-LOK^®^, Globus Medical, Audubon, Pennsylvania, USA), and in another case, lumbopelvic stabilization was performed. In the remaining cases where screw loosening was asymptomatic no specific treatment was necessary. Routine microbiological analyses of the explanted screws yielded negative results, indicating that mechanical factors were the likely cause of the loosening.

Multiple regression analyses were conducted to assess potential risk factors for screw loosening, but none of the following parameters reached statistical significance: overlapping screw wavers, FFP type of fracture, presence of a H-shaped fracture, presence of a comminuted fracture, and screw localization.

## Discussion

In this study, we present our results from four years of experience using the 3D-navigated monoportal PSS for fragility fractures of the posterior and/or anterior pelvic ring. This technique, utilizing iCT and a computer navigation system, represents an evolution and refinement in the field of minimally invasive osteosynthesis techniques. The advantages of this technique include the ability to safely place screws in the S1 level, as well as within the narrow pathways of the S2 level of the sacrum and the ramus of the pubic bone, without causing harm to adjacent sensitive tissue. All screws could be reliably implanted along the preoperatively intended trajectory, while still allowing intraoperative flexibility to accommodate changes in strategy and the need for additional screws. A considerable number of the treated fractures exhibited signs of dislocation (FFP3&4), but low-energy fractures did not require repositioning maneuvers. Preoperative secondary dislocation of fractures did not occur. However, by generating the 3D dataset immediately prior to trajectory planning and screw implantation without further patient mobilization, it would be possible to adapt to changing anatomical situations. The overall complication profile was very low with no instances of nerve root or vessel injury, and excellent results in terms of incision-to-suture time and blood loss, both of which are crucial factors for the clinical outcome of geriatric patients. However, in the long-term follow-up, we observed a considerable rate of screw loosening, which necessitated revision surgery in a several cases. Secondary escalation of the surgical strategy in terms of spinopelvic fixation due to insufficient healing of the fragility fracture was required in one case only.

In view of the experienced hardware failure, cement augmentation must be discussed as an alternative minimally invasive treatment for fragility fractures. Various procedures, such as balloon sacroplasty, radiofrequency sacroplasty, and cement sacroplasty, are available and have shown to be feasible treatment options^18–21^. However, it is important to note that vertebro-sacroplasty, in particular, carries a significant risk of cement leakage. Varying data in the literature, reporting cement leakage rates ranging from 0.4 to 24%, indicate a highly user-specific complication profile^22,23^. Moreover, cement augmentation is not recommended for dislocated sacral fractures.

Osteosynthesis of sacral fractures using sacroiliac screws under guidance of 3D computer navigation has shown potential as a superior alternative to conventional 2D-guided minimally invasive techniques^10,24^. Given the inter-individual variations in the spatial anatomical features of the sacrum, which can result in potentially narrow trajectories for screw implantation, 3D navigation provides an additional safety factor. This is especially relevant for transsacral screws, which must navigate the sacral neuroforamina on both sides within the same trajectory. As mentioned earlier, in addition to the advantages of 3D navigation, transsacral screws have demonstrated superior stability values in a finite element model of sacral fractures when compared to regular sacroiliac screws. It is therefore advisable to consider fixation with a single sacroiliac screw in both the S1 and S2 segments. Regardless of the type of sacroiliac screw used, if only one screw can be implanted, fixation in the S2 segment appears to be preferable over fixation in the S1 segment^11,12,25^. Based on these findings, we opted to implant S2 screws whenever the narrow anatomy of the trans-sacral corridor allowed for it.

In summary, minimally invasive 3D-navigated osteosynthesis of fragility fractures of the pelvis appears to be a promising approach, particularly in geriatric patients. However, the published data of its clinical application is limited, and the optimal fragility fractures of the pelvis is yet to be determined^7^. Therefore, our intention was to provide an illustrated description of this promising surgical technique and report our four years of clinical experience using it.

The technical feasibility of our setup, which involved intraoperatively acquired CT scans and a well-established 3D navigation system, was confirmed by the safe and precise implantation of screws, as well as the relatively short time from skin incision to wound closure and minimal blood loss. Another research group investigating this technique using alternative imaging and navigation devices also achieved consistently accurate screw positioning and a low perioperative complication profile^26^.

The arrangement of the technical devices in the operating theatre and the crucial surgical steps were integrated seamlessly into the routine workflow of our department. However, we acknowledge several potential disadvantages of the presented technique and the devices used. These include the cost of both implementing an intraoperative CT and 3D navigation system, which may limit its availability in certain hospitals or healthcare settings. Additionally, the higher radiation dosage associated with intraoperative CT scans compared to conventional procedures must be carefully considered. Proper radiation safety measures should be implemented to minimize potential risks associated with radiation exposure for both patients and surgical staff.

In our operative setting, all staff leave the operating theatre during the brief phase of acquiring the CT data set, ensuring no radiation exposure for the staff. Similar considerations apply for modern 3D fluoroscopy scanners, which use comparable or even higher radiation dosages^27^. Compared to these, the advantage of CT with its larger field of view becomes particularly apparent in 3D-navigated surgery of the spinopelvic region, especially in obese patients.

It is also important to note that the time consumption associated with intraoperative CT should be evaluated in the context of the specific procedure. Factors such as set-up time, image calculation time, and the need to briefly leave the operating room during acquisition can contribute to the overall time consumption. On the other hand, technically evolved intraoperative CT-based navigation systems offer precise real-time guidance and visualization, which can enhance surgical accuracy and potentially reduce the time required for procedures that particularly precise implant positioning^28^.

The peri- and postoperative complication rates were found to be excellent, and the intended early postoperative mobilization of patients was achieved in nearly all cases. Acknowledging the limitations of a small study cohort, it is important to note that general complications, including postoperative hematomas and wound healing disorders, are ultimately unavoidable. Nonetheless, the demonstrated minimally invasive approach shows its merits in terms of a low complication rate. One limitation of the minimally invasive approach could be primary or secondary dislocation of the pelvic ring fracture, which is more common in high impact trauma. However, due to the low-energy nature of fragility fractures of the sacrum and the typically intact stabilizing ligamentous apparatus, severe dislocations of these types of fractures are uncommon^29,30^. In case where the fracture is close to the symphysis or involves a dislocation of the symphysis gap, the safe implantation of a superior ramus screw into the pubic bone may not be feasible, and an additional open procedure may be necessary.

Another limitation of the presented study is the absence of a control group using alternative surgical techniques. Screw loosening occurred some cases with the studied technique, but the need for reoperation was low and consistent with previously published data. In a comparative study of different osteosynthesis techniques, Eckardt et al. found a good functional outcome, similar to our results, and comparable screw loosening rates, resulting in a reoperation rate of 9%. This showed a marked, yet non-significant superiority over a reoperation rate of 26% in the group of patients treated with regular sacroiliac screws^14^. Additional larger case series are needed for a more precise estimation of loosening rates. Regression analyses of relevant parameters in our patient cohort failed to identify significant risk factors. Given this context, we must assume that our sample size is too small to accurately assess causative risk factors. One possible cause of screw loosening to consider, is the particularly high biomechanical stress on a screw osteosynthesis bridging both sacroiliac joints, exposing it to opposing nutation forces. However, the fixation of transsacral screws in a total of four layers of cortical bone is considered protective against screw pullout or loosening. In individuals with osteoporosis and compromised bone quality in the sacral body, the compression applied to the sacroiliac joint and the sacral fracture may be reduced^31^. This reduced compression decreases the resistance against the vertical force that occurs during mobilization and may pose a vulnerability for implant loosening. In a recent biomechanical study of osteosynthesis of fragility fractures, Zderic et al. presented a prototype of screw-in-screw fixation in regular S1 screws and found lower implant movement compared to conventional S1 screws and comparable implant movement to transsacral screws^32^. Furthermore, Wagner et al. reported a screw loosening rate of only 4.7% with combined osteosynthesis using a transsacral bar and unilateral or bilateral sacroiliac screws^33^. Further studies on these techniques are of great interest, aiming to improve patients' outcomes by minimizing implant loosening rates to a minimum.

## Conclusion

The minimally invasive monoportal PSS for fragility fractures of the sacrum, as presented here, is a feasible and promising technological advancement in surgical treatment strategies for such injuries. Further refinement of the technique should focus on achieving reduced implant loosening and subsequent reoperation rates. This necessitates additional studies on biomechanical properties, comparative analyzes to other techniques, and the optimization of patient and implant selection.

## Supplementary Information

Supplementary Table 1.

## Data Availability

All data generated or analysed during this study are included in this published article [and its Supplementary Information files]. The data of this work are part of the dissertation of Mr. Martin Naisan.

## References

[1] Nanninga GL, de Leur K, Panneman MJ, van der Elst M, Hartholt KA (2014). Increasing rates of pelvic fractures among older adults: The Netherlands, 1986–2011. Age Ageing.

[2] Sullivan MP, Baldwin KD, Donegan DJ, Mehta S, Ahn J (2014). Geriatric fractures about the hip: Divergent patterns in the proximal femur, acetabulum, and pelvis. Orthopedics.

[3] Balogh Z, King KL, Mackay P, McDougall D, Mackenzie S, Evans JA (2007). The epidemiology of pelvic ring fractures: A population-based study. J. Trauma.

[4] Benzinger P, Becker C, Kerse N, Bleibler F, Buchele G, Icks A (2013). Pelvic fracture rates in community-living people with and without disability and in residents of nursing homes. J. Am. Med. Dir. Assoc..

[5] Kannus P, Palvanen M, Niemi S, Parkkari J, Jarvinen M (2000). Epidemiology of osteoporotic pelvic fractures in elderly people in Finland: Sharp increase in 1970–1997 and alarming projections for the new millennium. Osteoporos Int..

[6] Kelsey JL, Prill MM, Keegan TH, Quesenberry CP, Sidney S (2005). Risk factors for pelvis fracture in older persons. Am. J. Epidemiol..

[7] Rommens PM, Wagner D, Hofmann A (2017). Minimal invasive surgical treatment of fragility fractures of the pelvis. Chirurgia (Bucur).

[8] Wagner D, Hofmann A, Kamer L, Sawaguchi T, Richards RG, Noser H (2018). Fragility fractures of the sacrum occur in elderly patients with severe loss of sacral bone mass. Arch. Orthop. Trauma Surg..

[9] Wagner D, Kamer L, Sawaguchi T, Richards RG, Noser H, Rommens PM (2016). Sacral bone mass distribution assessed by averaged three-dimensional CT models: Implications for pathogenesis and treatment of fragility fractures of the sacrum. J. Bone Joint Surg. Am..

[10] Thakkar SC, Thakkar RS, Sirisreetreerux N, Carrino JA, Shafiq B, Hasenboehler EA (2017). 2D versus 3D fluoroscopy-based navigation in posterior pelvic fixation: Review of the literature on current technology. Int. J. Comput. Assist. Radiol. Surg..

[11] Zhao Y, Li J, Wang D, Liu Y, Tan J, Zhang S (2012). Comparison of stability of two kinds of sacro-iliac screws in the fixation of bilateral sacral fractures in a finite element model. Injury.

[12] Zhao Y, Zhang S, Sun T, Wang D, Lian W, Tan J (2013). Mechanical comparison between lengthened and short sacroiliac screws in sacral fracture fixation: A finite element analysis. Orthop. Traumatol.-Surg. Res..

[13] Wagner D, Kamer L, Rommens PM, Sawaguchi T, Richards RG, Noser H (2014). 3D statistical modeling techniques to investigate the anatomy of the sacrum, its bone mass distribution, and the trans-sacral corridors. J. Orthop. Res..

[14] Eckardt H, Egger A, Hasler RM, Zech CJ, Vach W, Suhm N (2017). Good functional outcome in patients suffering fragility fractures of the pelvis treated with percutaneous screw stabilisation: Assessment of complications and factors influencing failure. Injury.

[15] Mehling I, Hessmann MH, Rommens PM (2012). Stabilization of fatigue fractures of the dorsal pelvis with a trans-sacral bar. Operative technique and outcome. Injury.

[16] Sanders D, Fox J, Starr A, Sathy A, Chao J (2016). Transsacral-transiliac screw stabilization: Effective for recalcitrant pain due to sacral insufficiency fracture. J. Orthop. Trauma.

[17] Rommens PM, Hofmann A (2013). Comprehensive classification of fragility fractures of the pelvic ring: Recommendations for surgical treatment. Injury.

[18] Frey ME, DePalma MJ, Cifu DX, Bhagia SM, Daitch JS (2007). Efficacy and safety of percutaneous sacroplasty for painful osteoporotic sacral insufficiency fractures: A prospective, multicenter trial. Spine (Phila Pa 1976).

[19] Andresen R, Radmer S, Wollny M, Andresen JR, Nissen U, Schober HC (2017). CT-guided cement sacroplasty (CSP) as pain therapy in non-dislocated insufficiency fractures. Eur. J. Orthop. Surg. Traumatol..

[20] Andresen R, Radmer S, Andresen JR, Wollny M, Nissen U, Schober HC (2019). Clinical improvement and cost-effectiveness of CT-guided radiofrequency sacroplasty (RFS) and cement sacroplasty (CSP)—A prospective randomised comparison OF methods. Z. Orthop. Unfall..

[21] Andresen JR, Radmer S, Prokop A, Schroder G, Schober HC, Andresen R (2022). Sacral fragility fractures: Risk factors and outcomes after cement sacroplasty. Orthopadie (Heidelb).

[22] Kortman K, Ortiz O, Miller T, Brook A, Tutton S, Mathis J (2013). Multicenter study to assess the efficacy and safety of sacroplasty in patients with osteoporotic sacral insufficiency fractures or pathologic sacral lesions. J. Neurointerv. Surg..

[23] Bastian JD, Keel MJ, Heini PF, Seidel U, Benneker LM (2012). Complications related to cement leakage in sacroplasty. Acta Orthop. Belg..

[24] Florio M, Capasso L, Olivi A, Vitiello C, Leone A, Liuzza F (2020). 3D—Navigated percutaneous screw fixation of pelvic ring injuries—A pilot study. Injury.

[25] Zhao Y, Zhang S, Sun T, Wang D, Lian W, Tan J (2013). Mechanical comparison between lengthened and short sacroiliac screws in sacral fracture fixation: A finite element analysis. Orthop. Traumatol. Surg. Res..

[26] Ciolli G, Caviglia D, Vitiello C, Lucchesi S, Pinelli C, De Mauro D (2021). Navigated percutaneous screw fixation of the pelvis with O-arm 2: Two years' experience. Med. Glas (Zenica).

[27] Malham GM, Wells-Quinn T (2019). What should my hospital buy next?-Guidelines for the acquisition and application of imaging, navigation, and robotics for spine surgery. J. Spine Surg..

[28] Otomo N, Funao H, Yamanouchi K, Isogai N, Ishii K (2022). Computed tomography-based navigation system in current spine surgery: A narrative review. Medicina (Kaunas).

[29] Gross A, Kuttner H, Shariat K, Benninger E, Meier C (2022). The surgical management of highly unstable fragility fractures of the sacrum with spinopelvic dissociation: A case series and proposal of a surgical treatment algorithm. Injury-Int. J. Care Inj..

[30] Wagner D, Ossendorf C, Gruszka D, Hofmann A, Rommens PM (2015). Fragility fractures of the sacrum: How to identify and when to treat surgically?. Eur. J. Trauma Emerg. Surg..

[31] Ziran N, Collinge CA, Smith W, Matta JM (2022). Trans-sacral screw fixation of posterior pelvic ring injuries: Review and expert opinion. Patient Saf. Surg..

[32] Zderic I, Wagner D, Schopper C, Lodde M, Richards G, Gueorguiev B (2021). Screw-in-screw fixation of fragility sacrum fractures provides high stability without loosening-biomechanical evaluation of a new concept. J. Orthop. Res..

[33] Wagner D, Kisilak M, Porcheron G, Kramer S, Mehling I, Hofmann A (2021). Trans-sacral bar osteosynthesis provides low mortality and high mobility in patients with fragility fractures of the pelvis. Sci. Rep..

